# Legal Notes for Institutional Workers

**Published:** 1917-04-21

**Authors:** 


### LEGAL NOTES FOR INSTITUTIONAL WORKERS.

### Warranties in Contracts.

Commenting on the recent case of *Humphries* v. *Miller*, the *Solicitors' Journal* says: "Our Courts nowadays lean against the introduction of implied warranties into contracts." Such warranties have a recognised place in certain contracts, but, the writer proceeds, "novel warranties not known to the law or recognised by the authoritative text-book writers, however just and reasonable they may seem, are not regarded with sympathy by our judges." The point of the case turned on the contention of the plaintiff that any person who takes rooms for another in a boarding-house impliedly warrants the tenant to be a fit and proper person to reside therein. A patient suffering from leprosy and his daughter took rooms in a boarding-house; they disclosed the fact that the patient was in ill-health, but not the disease or its contagious character. The patient's doctor, indeed, according to the jury's finding, had expressly stated to the proprietress that the illness was not due to any infectious disease. Both the patient and the doctor, but not the patient's daughter, were aware that the disease was leprosy, and it is not surprising that a common jury found for the plaintiff, the proprietress, with very substantial damages. But a cause of action which the law will recognise is not easy to find. The plaintiff sued the daughter and the doctor—the patient having died—alleging (1) breach of warranty, (2) deceit, and (3) conspiracy. The second and third grounds of action, which sound in tort, evidently fail, at least so far as the female defendant is concerned, because she did not know that her father was a leper. The doctor could not conspire with himself, so that conspiracy goes in his case too, and, further, as he was under no duty to disclose the nature of his patient's disease, an action resting on common-law fraud—*i.e.* deceit—fails. But was there not an implied warranty to disclose the infectious or contagious nature of the disease on the part of the patient, whose daughter was also his executrix, and the doctor? The Court of Appeal, as well as the judge of first instance, who entered judgment for the defendants in the face of the jury's verdict, declined to read into a boarding-house tenancy any such implied warranty. The landlord who lets furnished apartments warrants their fitness for habitation by the tenant, but there is no reciprocal warranty by him of his fitness to inhabit them.

In the South African case, *Oates* v. *Niland* (S. Afr. L.R. 1914, C.P.D.), the plaintiff (a dentist) brought his action for dental attendance and a plate supplied to the defendant. The plaintiff, who lived at a considerable distance from the defendant, had made for him a plate containing a certain number of teeth. During the period of over a year no complaint whatever was made by the defendant that the teeth were not fitting, and no notice of any dissatisfaction with the plate was given to the plaintiff. Even after this time had elapsed he did not repudiate the contract, and the teeth showed signs of having been considerably used. His statement was that he found the plate painful to wear, that he tried it from time to time at short intervals, until, finding it always uncomfortable, he ceased altogether to wear it. As, however, he did not give the plaintiff notice of the insufficiency of the plate and teeth, the Court held that it was too late for him to set up as a defence to an action for the price that they were unsuitable.

